# PK/PD Modeling of the PDE7 Inhibitor—GRMS-55 in a Mouse Model of Autoimmune Hepatitis

**DOI:** 10.3390/pharmaceutics13050597

**Published:** 2021-04-21

**Authors:** Artur Świerczek, Hanna Plutecka, Marietta Ślusarczyk, Grażyna Chłoń-Rzepa, Elżbieta Wyska

**Affiliations:** 1 Department of Pharmacokinetics and Physical Pharmacy, Faculty of Pharmacy, Jagiellonian University Medical College, 9 Medyczna Street, 30-688 Kraków, Poland; 2 Department of Internal Medicine, Faculty of Medicine, Jagiellonian University Medical College, 8 Skawińska Street, 31-066 Kraków, Poland; hanka.plutecka@uj.edu.pl; 3 Department of Medicinal Chemistry, Faculty of Pharmacy, Jagiellonian University Medical College, 9 Medyczna Street, 30-688 Kraków, Poland; marietta.slusarczyk@doctoral.uj.edu.pl (M.Ś.); grazyna.chlon-rzepa@uj.edu.pl (G.C.-R.)

**Keywords:** disease progression modeling, phosphodiesterase inhibitors, cytokines, concanavalin A-induced hepatitis

## Abstract

This study aimed to assess the efficacy and explore the mechanisms of action of a potent phosphodiesterase (PDE)7A and a moderate PDE4B inhibitor GRMS-55 in a mouse model of autoimmune hepatitis (AIH). The concentrations of GRMS-55 and relevant biomarkers were measured in the serum of BALB/c mice with concanavalin A (ConA)-induced hepatitis administered with GRMS-55 at two dose levels. A semi-mechanistic PK/PD/disease progression model describing the time courses of measured biomarkers was developed. The emetogenicity as a potential side effect of the studied compound was evaluated in the α_2_-adrenoceptor agonist-induced anesthesia model. The results indicate that liver damage observed in mice challenged with ConA was mainly mediated by TNF-α and IFN-γ. GRMS-55 decreased the levels of pro-inflammatory mediators and the transaminase activities in the serum of mice with AIH. The anti-inflammatory properties of GRMS-55, resulting mainly from PDE7A inhibition, led to a high hepatoprotective activity in mice with AIH, which was mediated by an inhibition of pro-inflammatory signaling. GRMS-55 did not induce the emetic-like behavior. The developed PK/PD/disease progression model may be used in future studies to assess the potency and explore the mechanisms of action of new investigational compounds for the treatment of AIH.

## 1. Introduction

Autoimmune hepatitis (AIH) is caused by the loss of immune tolerance to self-antigens expressed in liver cells and leads to progressive damage of this organ [1]. AIH is quite a rare condition that affects predominantly females, but it may also occur in males [1,2]. If not treated efficiently, this disorder can result in cirrhosis and liver failure, which is one of the indications for orthotopic liver transplantation [3]. The standard treatment for AIH is immunosuppressive drugs, such as azathioprine, administered along with corticosteroids [4]. However, the use of these medications is associated with adverse effects. For example, azathioprine may not only cause leukopenia and increase the susceptibility of an individual to infectious diseases, but can also cause cholestasis and liver damage. In turn, administration of corticosteroids may lead to steroidal myopathy, osteoporosis, diabetes, obesity, and thromboembolic syndrome. Despite many years of research focusing on finding new, safer, and more effective therapeutics for AIH, immunosuppressive therapy is still most commonly used for the treatment of this condition [4].

In recent times, phosphodiesterase (PDE) inhibitors have been extensively studied as a potential option to treat inflammatory and autoimmune disorders. In particular, selective PDE4 inhibitors [5], PDE7 inhibitors, dual PDE4/7 inhibitors [6,7], and non-selective PDE inhibitors [8] are synthesized and their efficacy in the treatment of these conditions is studied. PDE inhibitors exhibit anti-inflammatory activity by decreasing the levels of pro-inflammatory mediators and increasing the levels of anti-inflammatory cytokines, which are key players in the pathogenesis of inflammatory and autoimmune diseases. Among them, PDE4 and PDE7 inhibitors function through elevating the intracellular concentration of the second messenger, cyclic AMP (cAMP), in immune cells [8]. It was observed that compounds such as PDE7 inhibitors may additionally influence the proliferation of T cells, and thus exhibit an immunomodulatory effect [9]. In our previous studies, we revealed that GRMS-55, a strong PDE7A inhibitor with moderate PDE4B-inhibitory properties, can effectively ameliorate the symptoms of collagen-induced arthritis and LPS-induced endotoxemia in rats [10,11]. We also reported that this compound decreased the levels of TNF-α and activities of transaminases in immune-mediated ConA-induced hepatitis in mice, a commonly used animal model of AIH. This AIH model is characterized by fulminant liver damage associated with excessive production and release of several inflammatory mediators, among which TNF-α, IL-6, IL-10, and IFN-γ seem to be influencing the disease severity [12,13]. In contrast to other animal models of acute immune hepatic injury, ConA-induced liver damage is primarily driven by the activation and recruitment of T cells. Therefore, it resembles many features of immune-mediated hepatitis in humans, such as AIH, or acute viral hepatitis, and is listed among the most frequently used models of human AIH [14]. Specifically, as ConA-induced damage is severe and acute and regresses upon ConA elimination from the organism, it may be considered as a model of acute-onset AIH in humans, which is often observed at the beginning of this disorder or during relapses [2]. Fas-mediated apoptosis has been shown to be an important factor contributing to liver cell damage in this model [15]. In addition, necrosis of hepatocytes was observed in ConA-treated mice [12]. Similarly, both necrosis and Fas-mediated apoptosis of hepatocytes were reported in human AIH [16,17]. Previous studies on ConA-induced hepatitis models carried out by other research groups focused on assessing the dose–response relationship rather than the concentration–effect relationship of investigated compounds [18,19,20]. The possible reason for this gap was the lack of PK/PD models that would allow correlating the drug concentration–effect data and performing a quantitative assessment of the efficacy of studied compounds.

The present study aimed to assess the pharmacological activity of the PDE inhibitor, GRMS-55, and elucidate its mechanisms of action in ConA-induced hepatitis in mice by correlating the compound concentration–effect data using a PK/PD/disease progression model designed for this purpose. In addition, the study analyzed the mechanisms involved in the development of ConA-induced hepatitis. To this end, we investigated the time courses of GRMS-55 concentrations, changes in time of several inflammatory mediators (TNF-α, IFN-γ, IL-10, and IL-6), and biomarkers of liver damage (alanine aminotransferase (ALT) and aspartate aminotransferase (AST)) in the serum of animals that were pretreated with GRMS-55 and subsequently challenged with ConA. Based on the data obtained, we developed a semi-mechanistic PK/PD/disease progression model to provide a deeper insight into the mechanism of action of GRMS-55 and disease development in a mouse model of AIH. Furthermore, the influence of the studied compound on the levels of several other relevant cytokines, such as IL-1β, IL-4, IL-12, and IL-17A, was investigated in mice with ConA-induced hepatitis. Finally, considering the fact that the clinical utility of PDE4 inhibitors is significantly limited by their gastrointestinal side effects, such as nausea and vomiting, the effect of GRMS-55 dosing on the duration of anesthesia induced by α_2_-adrenoceptor agonist administration in mice was investigated. This animal model is a well-established surrogate model of PDE4 inhibitor-triggered nausea and emesis in humans [21].

## 2. Materials and Methods

### 2.1. Materials

DMSO and polyethylene glycol (PEG) 400 were purchased from Sigma-Aldrich (Steinheim, Germany). ConA was obtained from Santa Cruz Biotechnology (Dallas, TX, USA). Rolipram and BRL-50481 were purchased from Cayman Chemical (Ann Arbor, MI, USA). GRMS-55 (4-(1,3-dimethyl-2,6-dioxo-8-(phenethylamino)-1,2,3,6-tetrahydro-7*H*-purin-7-yl)-*N*′-(2-hydroxybenzylidene)butanehydrazide) and 4-(8-((furan-2-ylmethyl)amino)-1,3-dimethyl-2,6-dioxo-1,2,3,6-tetrahydro-7*H*-purin-7-yl)-*N*′-(2-hydroxybenzylidene)butanehydrazide were obtained from the Department of Medicinal Chemistry, Faculty of Pharmacy, Jagiellonian University Medical College (Kraków, Poland). Ketamine and xylazine were from Biowet (Puławy, Poland). Other reagents used in the studies were purchased from Merck (Darmstadt, Germany).

### 2.2. Animals

Female BALB/c mice at 2–3 months of age weighing 18–23 g (Mossakowski Medical Research Center, Polish Academy of Sciences, Warszawa, Poland) and male CD-1 mice at 2 months of age weighing 18–23 g (Animal Facility at the Faculty of Pharmacy, Jagiellonian University Medical College, Kraków, Poland) were used in the experiments. Animals were kept under a 12:12 h light–dark cycle and constant temperature of 22 °C. They had free access to food and water and were fasted for 8–12 h prior to the experiments. All animal procedures were approved by the First Local Ethical Committee on Animal Testing in Kraków (No. 206/2018 approved on 19 December 2018 and 424/2020 approved on 30 September 2020). All applicable international, national, and institutional guidelines for the care and use of animals were followed.

### 2.3. Experimental Procedures

#### 2.3.1. Pharmacokinetic Study

Pharmacokinetics of GRMS-55 was evaluated in female BALB/c mice following intraperitoneal (IP) administration of this compound at doses of 50 and 100 mg·kg^−1^. The investigated compound was dissolved in a vehicle composed of DMSO, PEG 400, and water for injection at a ratio of 25:25:50 (*v*/*v*/*v*). Animals were exsanguinated at 5, 15, 30, 60, 90, and 120 min following GRMS-55 administration. Blood samples were collected, allowed to clot for 30 min at room temperature, and centrifuged at 3000× *g* for 10 min. The serum was separated and stored at −20 °C until analysis.

#### 2.3.2. ConA-induced Hepatitis and Compound Administration

ConA was dissolved in sterile saline (2 mg·mL^−1^). The studied compounds (GRMS-55, rolipram, and BRL-50481) were dissolved in a mixture of DMSO, PEG 400, and water for injection at a ratio of 25:25:50 (*v*/*v*/*v*). Mice were given an IP injection of the investigated compounds or the corresponding volume of vehicle alone and, thirty minutes later, intravenously (IV) administered with ConA solution. An additional control group received vehicle alone IP instead of the studied compounds followed by IV injection of sterile saline. ConA was given at a dose of 20 mg·kg^−1^. Mice were exsanguinated at several time points post-ConA injection. Blood samples were allowed to clot for 0.5 h at room temperature and then centrifuged at 3000× *g* for 10 min to obtain the serum, which was stored at −20 °C until further analysis.

#### 2.3.3. Assessment of Emetogenicity

Male CD-1 mice were anesthetized with xylazine (10 mg·kg^−1^) and ketamine (80 mg·kg^−1^) in saline for injection-administered IP. Fifteen minutes later, the animals were IP-administered with GRMS-55 at doses of 50 or 100 mg·kg^−1^, rolipram at a dose of 10 mg·kg^−1^, or the corresponding volume of vehicle and subsequently placed in a dorsal recumbency position. The return of the righting reflex (i.e., when the animal turned itself spontaneously to a prone position) was used as an endpoint to measure the duration of anesthesia. In this test, shortening of the sleeping time is a behavioral correlate of emesis.

#### 2.3.4. Experimental Design and Sample Collection

The influence of PDE inhibitors on the levels of inflammatory mediators and transaminases in ConA-treated mice was investigated in two separate experiments. In the preliminary study, the animals were pretreated IP with GRMS-55 at a dose of 50 mg·kg^−1^, the PDE4-selective inhibitor rolipram at a dose of 10 mg·kg^−1^, or the PDE7-selective inhibitor BRL-50481 at a dose of 50 mg·kg^−1^. Doses of the investigated compounds were chosen based on the results of previous studies [10,11]. Subsequently, mice were given an IV injection of ConA and sacrificed at 8 h following mitogen dosing. The levels of TNF-α, IL-6, and IFN-γ and the activities of ALT and AST in the mouse serum were measured. This preliminary experiment allowed comparing the activity of the studied PDE inhibitors in this AIH model and helped to select the doses of GRMS-55 for a subsequent pharmacodynamic study. In the final pharmacodynamic experiment, GRMS-55 was administered at two dose levels (50 and 100 mg·kg^−1^) followed by ConA administration at a dose of 20 mg·kg^−1^. Animals were sacrificed at various time points post-ConA dosing (1, 2, 4, 8 and 24 h). The levels of TNF-α, IL-1β, IL-4, IL-6, IL-10, IL-12, IL-17A, and IFN-γ were measured using a multiplex Luminex assay. In addition, the activities of ALT and AST in the mouse serum were assessed. The concentration–time profiles of the selected biomarkers obtained in this experiment combined with the results of pharmacokinetic analysis allowed the development of a semi-mechanistic PK/PD/disease progression model and the investigation of concentration–effect relationships of GRMS-55.

### 2.4. Analytical Methods

To analyze GRMS-55 concentrations in the mouse serum, we used the previously developed HPLC method with UV detection. A detailed description of this method was published in a supplementary material of our previous publication [10]. Briefly, 10 μL of 4-(8-((furan-2-ylmethyl)amino)-1,3-dimethyl-2,6-dioxo-1,2,3,6-tetrahydro-7*H*-purin-7-yl)-*N*′-(2-hydroxybenzylidene)butanehydrazide solution as internal standard (IS) was added to tubes containing 100 μL of the analyzed serum. The samples were mixed with 20 μL of 1 M hydrochloric acid solution, subjected to extraction with 1 mL of dichloromethane, and centrifuged at 10,000× *g* for 8 min. The organic layers were transferred to new tubes and evaporated with nitrogen at 37 °C. Dry residues were then dissolved in 100 μL of mobile phase and transferred to glass vial inserts for HPLC analysis (LaChrom Elite, Merck-Hitachi, Darmstadt, Germany). The separation was achieved on a LiChrospher 100 RP-18 (250 × 4 mm) column (particle size of 5 μm) protected with a LiChroCART guard column (Merck, Darmstadt, Germany) using the mobile phase composed of 20 mM solution of KH_2_PO_4_ (pH = 4.5) and acetonitrile at a ratio of 45:55 (*v*/*v*) pumped at a flow rate of 1 mL min^−1^ at ambient temperature. The analytical wavelength was set to 209 nm. In these conditions, the retention time of GRMS-55 was 9.2 min and for IS it was 5.3 min. The analytical method was validated according to FDA Bioanalytical Method Validation, Guidance for Industry [22]. Specificity of the method was estimated by analyzing six different independent samples of the blank serum. No interfering peaks of endogenous serum components at the retention time of GRMS-55 and IS were observed in the chromatograms. The calibration curve was constructed by plotting the ratios of the peak area of the studied compound to IS vs. concentrations of GRMS-55 and it was linear in the tested concentration range, i.e., 0.050–20 mg·L^−1^. Within runs and between runs, precision and accuracy met the acceptance criteria, i.e., they were less than 15% expressed as coefficients of variation % (CVs%) or ±15% of the nominal concentrations, respectively, for all quality control levels. Recoveries of the analyzed compound and IS assessed by comparing the peak areas of extracted samples with corresponding extracts of blanks spiked with the analyte post-extraction ranged from 94% to 98%. The lower limit of quantification was 0.05 mg·L^−1^. GRMS-55 in serum samples was stable at room temperature for at least 12 h and at –20 °C for a period of 12 months. The stock solutions were stable for over one month at 4 °C.

The serum samples from the preliminary pharmacological study were analyzed using ELISA tests according to the protocols provided by the manufacturers. The TNF-α concentrations were measured using the Mouse TNF-α Quantikine ELISA Kit (R&D Systems, Minneapolis, MN, USA), while the concentrations of IL-6 and IFN-gamma were quantified by ELISA kits manufactured by Cloud-Clone Corp. (Houston, TX, USA). The absorbance of samples was measured on a POLARstar Omega multi-mode microplate reader (BMG LABTECH, Ortenberg, Germany) set at 450 nm wavelength. TNF-α, IL-1β, IL-6, IL-4, IL-10, IL-12, IL-17A, and IFN-γ serum levels in samples from pharmacodynamic experiment were determined by a premixed Magnetic Luminex Assay (R&D Systems, Inc., Minneapolis, MN, USA) according to the manufacturer’s protocol. Serum AST and ALT activities were quantified on a chemistry analyzer BS-800 (Shenzhen Mindray Bio-medical Electronics Co., Ltd., Shenzhen, China).

### 2.5. Data and Statistical Analysis

#### 2.5.1. Pharmacokinetics and PK/PD/Disease Progression Modeling

Initially, non-compartmental analysis of the concentration–time data of GRMS-55 was performed. Subsequently, pharmacokinetic modeling was conducted. Different compartmental models with linear and non-linear elimination were tested, and the final pharmacokinetic model with the best goodness-of-fit criteria was chosen. Several measures of goodness-of-fit were evaluated, i.e., value of the objective function, Akaike Information Criterion, Bayesian Information Criterion, visual inspection of fitting, and a precision of parameter estimates expressed as CVs%. Pharmacokinetic model parameters were estimated using the ADAPT 5 software (BMSR, Los Angeles, CA, USA) based on the weighted least squares algorithm.

**Figure 1:**
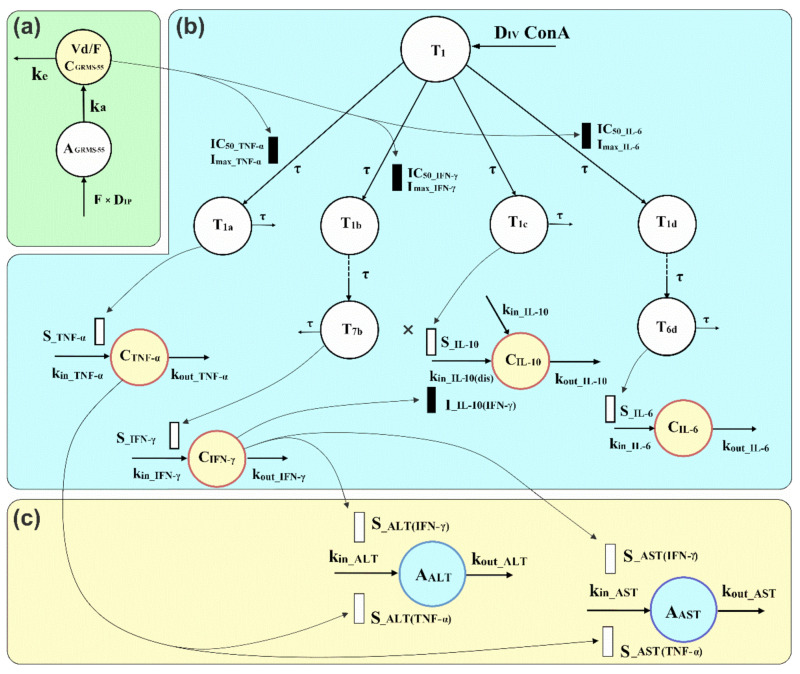
Schematic representation of the proposed PK/PD/disease progression model to describe the changes in the concentrations of TNF-α, IFN-γ, IL-6, and IL-10 (C_TNF-α_, C_IFN-γ_, C_IL-6_, and C_IL-10_) and activities of ALT and AST (A_ALT_ and A_AST_) in the serum of female BALB/c mice pretreated IP with GRMS-55 at two dose levels or vehicle alone and subsequently challenged with ConA. The model is composed of (**a**) pharmacokinetic, (**b**) pharmacodynamic, and (**c**) disease progression parts; T_1_ is the amount of a hypothetical precursor of production of inflammatory mediators; T_1a_, T_1b_–T_7b_, T_1c_, and T_1d_-T_6d_ are quantities of hypothetical precursors of TNF-α, IFN-γ, IL-10, and IL-6, respectively, participating in the synthesis and/or release of these inflammatory mediators; I_max___TNF-α_, I_max___IFN-γ_, and I_max___IL-6_ are the maximal inhibitory capacities of GRMS-55 on the production of mediators; C_GRMS-55_ is the concentration of the test compound in the serum, A_GRMS-55_ is its amount at the site of absorption, and F is the bioavailability of GRMS-55 following IP administration, Vd/F is the apparent volume of distribution of the test compound following IP dosing, k_a_ and k_e_ are the first-order rate constants of absorption and elimination of the studied compound, respectively; IC_50___TNF-α_, IC_50___IFN-γ_, and IC_50___IL-6_ are the concentrations of GRMS-55 producing 50% of the maximal inhibition of each mediator production, τ is the mean transit and disappearance from the system time of precursors of inflammatory mediators; k_in___TNF-α_, k_in___IFN-γ_, k_in___IL-6_, k_in___IL-10_, k_in___ALT_, and k_in___AST_ are the zero-order rate constants of production of each biomarker; k_in___IL-10(dis)_ is the zero-order production rate constant of IL-10 at the disease state; k_out_TNF-α_, k_out_IFN-γ_, k_out_IL-6_, k_out_IL-10_, k_out_ALT_, and k_out_AST_ are the first-order rate constants of each biomarker loss; S__TNF-α_, S__IFN-γ_, S__IL-6_, and S__IL-10_ are, specific for each mediator, stimulation coefficients accounting for the amplification of the production of inflammatory mediators in response to ConA challenge; S__ALT(IFN-γ)_ and S__ALT(TNF-α)_ are stimulation coefficients describing the increase in ALT activity in the serum induced by IFN-γ and TNF-α, while S__AST(IFN-γ)_ and S__AST(TNF-α)_ are the stimulation coefficients accounting for amplification of AST release induced by IFN-γ and TNF-α; I__IL-10(IFN-γ)_ is the linear inhibitory coefficient accounting for the inhibition of IL-10 production by IFN-γ.

A PK/PD/disease progression model illustrated in Figure 1 was developed considering the mechanism of action of the investigated compound and the mechanisms of progression of ConA-induced hepatitis, i.e., the subsequent stages leading to the production and release of relevant biomarkers.

Moreover, possible interrelationships and interactions among the measured biomarkers were considered during the model building process. The model is composed of indirect response models as well as transit compartment models that account for the delay observed between ConA administration and the release of relevant biomarkers of inflammation and liver damage. In addition, this approach enabled capturing the delay between GRMS-55 dosing and its pharmacological effect, resulting from the multi-stage mechanism of action of this compound. The pharmacodynamic part of the PK/PD/disease progression model, shown in Figure 1b, is composed of four series of transit compartments, which have the same origin at the first transit compartment of the model (T_1_). This part accounts for the production of four inflammatory mediators, namely TNF-α, IFN-γ, IL-6, and IL-10. The number of transit compartments for each series was determined by a trial-and-error method. Since ALT and AST activities in the serum are directly related to the extent of liver damage, it was assumed that their time courses reflect the hepatitis progression in mice. The part of the model describing AIH progression (Figure 1c) consists of two separate indirect response models to describe ALT and AST turnover, in which increases in the TNF-α and IFN-γ concentrations are driving forces accounting for the rise in transaminase levels. Alternative models were considered and tested, assuming a possible direct influence of GRMS-55 on the production of transaminases or engagement of IL-6 and IL-10 in ALT and AST turnover, but the model assuming the exclusive contribution of TNF-α and IFN-γ to ALT and AST elevations exhibited the most favorable goodness-of-fit criteria and best captured the experimental data. The parameter estimation was performed in a sequential manner. Firstly, the estimated values of pharmacokinetic parameters were fixed during PK/PD model fitting, and then pharmacodynamic parameters were fixed in the final run of the PK/PD/disease progression model. As a result, one single set of pharmacokinetic, pharmacodynamic, and disease progression parameters was obtained. Modeling was conducted in ADAPT 5 software using naïve-pooled data and the weighted least squares algorithm.

The model presented in Figure 1 is based on the assumption that the test PDE inhibitor acts in the initial phase of hepatitis development, when the levels of the majority of the investigated biomarkers are not yet elevated in the blood. This is in agreement with a multi-stage mechanism of action of PDE4 and PDE7 inhibitors, which are known to inhibit degradation of the intracellular second messenger, cAMP, in immune cells. cAMP subsequently phosphorylates protein kinase A (PKA), which interacts with various transcription factors, thereby regulating the expression of genes of inflammatory mediators [6]. Pharmacokinetic parameters of GRMS-55 (Equations (1) and (2)) were estimated prior to the PK/PD/disease progression analyses, based on the following differential equations:

(1) $$\frac{{\mathrm{dA}}_{\mathrm{GRMS}-55}}{dt}=-k_{a}\times A_{\mathrm{GRMS}-55},{\;A}_{\mathrm{GRMS}-55}\left(0\right)=F\times D_{\mathrm{IP}},$$

(2) $$\frac{{\mathrm{dX}}_{\mathrm{GRMS}-55}}{dt}=k_{a}\times A_{\mathrm{GRMS}-55}-k_{e}\times X_{\mathrm{GRMS}-55},{\;X}_{\mathrm{GRMS}-55}\left(0\right)=0$$

where A_GRMS-55_ and X_GRMS-55_ are the amounts of GRMS-55 at the site of absorption and in the central compartment, respectively. k_a_ and k_e_ are the first-order rate constants of absorption and elimination of the studied compound. The output values of Equation (2) were divided by the apparent volume of distribution of GRMS-55 following IP administration (Vd/F_-_); thus, the predicted by the model values represent the serum concentrations of GRMS-55 (C_GRMS-55_ = X_GRMS-55_/Vd/F).

Pharmacodynamic parameters of the proposed PK/PD/disease progression model were obtained based on a set of differential equations. The first equation in this set (Equation (3)) describes the time course of the first hypothetical precursor of the production of inflammatory mediators, which is activated immediately following IV administration of ConA, at 0.5 h following GRMS-55 dosing:

(3) $$\frac{{\mathrm{dT}}_{1}}{dt}=\left\{\begin{array}{c} 0,\;\;\;for\;\;\;t<0.5,\;\;\;T_{1}\left(0\right)=0,\\ -T_{1}\times \frac{1}{\tau },\;\;\;for\;\;\;t\geq 0.5,\;\;\;T_{1}\left(0.5\right)=1,\; \end{array}\right.$$

Therefore, the initial condition of Equation (3) was set to 0 at time 0, while the condition at 0.5 h was arbitrarily fixed to 1 for simplicity, which corresponds to the initial amount of the hypothetical precursor in the first transit compartment.

The subsequent series of transit compartments of the pharmacodynamic part of the model correspond to the pathophysiological pathways leading to the overproduction of the relevant inflammatory mediators. The final equations of each pathway, i.e., Equations (5), (8), (11), and (13) are indirect response models accounting for TNF-α, IFN-γ, IL-6, and IL-10 turnover, respectively. The equations describing profiles of the studied cytokines and their hypothetical precursors are as follows:

for the TNF-α pathway:

(4) $$\frac{{\mathrm{dT}}_{1a}}{dt}=\left(T_{1}\times \left(1-\frac{I_{\max\_\mathrm{TNF}-\alpha }\times C_{\mathrm{GRMS}-55}}{{\mathrm{IC}}_{50\_\mathrm{TNF}-\alpha }+C_{\mathrm{GRMS}-55}}\right)-T_{1a}\right)\times \frac{1}{\tau },{\;T}_{1a}\left(0\right)=0,$$

(5) $$\;\frac{{\mathrm{dC}}_{\mathrm{TNF}-\alpha }}{dt}=(1+T_{1a}\times S{\_}_{\mathrm{TNF}-\alpha })\times k_{\mathrm{in}\_\mathrm{TNF}-\alpha }-k_{\mathrm{out}\_\mathrm{TNF}-\alpha }\times C_{\mathrm{TNF}-\alpha },{\;C}_{\mathrm{TNF}-\alpha }\left(0\right)=R_{0\_\mathrm{TNF}-\alpha },$$

for the IFN-γ pathway:

(6) $$\frac{{\mathrm{dT}}_{1b}}{dt}=\left(T_{1}\times \left(1-\frac{I_{\max\_\mathrm{IFN}-\gamma }\times C_{\mathrm{GRMS}-55}}{{\mathrm{IC}}_{50\_\mathrm{IFN}-\gamma }+C_{\mathrm{GRMS}-55}}\right)-T_{1b}\right)\times \frac{1}{\tau },{\;T}_{1b}\left(0\right)=0,$$

(7) $$\frac{{\mathrm{dT}}_{\left(n+1\right)b}}{\mathrm{dt}}=\left(T_{nb}-T_{\left(n+1\right)b}\right)\times \frac{1}{\tau },{\;T}_{\left(n+1\right)b}\left(0\right)=0,n=1\ldots 6,$$

(8) $$\frac{{\mathrm{dC}}_{\mathrm{IFN}-\gamma }}{dt}=(1+T_{7b}\times S{\_}_{\mathrm{IFN}-\gamma })\times k_{\mathrm{in}\_\mathrm{IFN}-\gamma }-k_{{\mathrm{out}}_{\mathrm{IFN}}-\gamma }\times C_{\mathrm{IFN}-\gamma },{\;C}_{\mathrm{IFN}-\gamma }\left(0\right)=R_{0\_\mathrm{IFN}-\gamma }$$

for the IL-6 pathway:

(9) $$\frac{{\mathrm{dT}}_{1d}}{dt}=\left(T_{1}\times \left(1-\frac{I_{\max\_\mathrm{IL}-6}\times C_{\mathrm{GRMS}-55}}{{\mathrm{IC}}_{50\_\mathrm{IL}-6}+C_{\mathrm{GRMS}-55}}\right)-T_{1d}\right)\times \frac{1}{\tau },{\;T}_{1d}\left(0\right)=0,$$

(10) $$\frac{{\mathrm{dT}}_{\left(n+1\right)d}}{dt}=\left(T_{nd}-T_{\left(n+1\right)d}\right)\times \frac{1}{\tau },{\;T}_{\left(n+1\right)d}\left(0\right)=0,n=1\ldots 5,$$

(11) $$\frac{{\mathrm{dC}}_{\mathrm{IL}-6}}{dt}=(1+T_{6d}\times S{\_}_{\mathrm{IL}-6})\times k_{\mathrm{in}\_\mathrm{IL}-6}-k_{{\mathrm{out}}_{\mathrm{IL}}-6}\times C_{\mathrm{IL}-6},{\;C}_{\mathrm{IL}-6}\left(0\right)=R_{0\_\mathrm{IL}-6},$$

and for the IL-10 pathway:

(12) $$\frac{{\mathrm{dT}}_{1c}}{dt}=\left(T_{1}-T_{1c}\right)\times \frac{1}{\tau },{\;T}_{1c}\left(0\right)=0,$$

(13) $$\frac{{\mathrm{dC}}_{\mathrm{IL}-10}}{dt}=\left\{\begin{array}{c} k_{\mathrm{in}\_\mathrm{IL}-10}-k_{{\mathrm{out}}_{\mathrm{IL}}-10}\times C_{\mathrm{IL}-10},\;\;\;for\;\;\;t<0.5,\\ k_{\mathrm{in}\_\mathrm{IL}-10}+F_{\mathrm{IL}-10}\times k_{\mathrm{in}\_\mathrm{IL}-10\left(\mathrm{dis}\right)}-k_{{\mathrm{out}}_{\mathrm{IL}}-10}\times C_{\mathrm{IL}-10},\;\;\;for\;t\geq 0.5, \end{array}\;\;\;\right.C_{\mathrm{IL}-10}\left(0\right)=R_{0\_\mathrm{IL}-10},$$

(14) $$F_{\mathrm{IL}-10}=1+T_{1c}\times S{\_}_{\mathrm{IL}-10}-C_{\mathrm{IFN}-\gamma }\times I{\_}_{\mathrm{IL}-10\left(\mathrm{IFN}-\gamma \right)}$$

It was noted that following ConA-dosing, a new IL-10 steady-state concentration was achieved in the serum of diseased animals, higher than the baseline observed in healthy mice. It was assumed that this phenomenon was caused by an increased rate of IL-10 synthesis due to inflammation. Thus, an additional zero-order production rate constant of IL-10 was introduced at time 0.5 h (k_in_IL-10(dis)_). In addition, it was assumed that the synthesis of IL-10 is simultaneously driven by ConA and inhibited by IFN-γ, while no direct effect of GRMS-55 on the production of IL-10 is present. This assumption is in line with the literature data, as an inhibition of IL-10 expression by IFN-γ as well as increase in IL-10 serum concentrations in response to ConA administration in mice were observed in previous studies [13,23]. Nonetheless, concurrent models assuming a direct effect of other cytokines and GRMS-55 on the production of IL-10 were tested as well, but the goodness-of-fit criteria supported the selection of the final model shown in Figure 1. In this model, the F_IL-10_ function, which operates on k_in_IL-10(dis)_, accounts for the combined effect of ConA and IFN-γ on the production of IL-10 in diseased animals. IFN-γ inhibits the production of IL-10 with the linear inhibitory coefficient (I__IL-10(INF-γ)_), while ConA, via the series of transit compartments (T_1_ and T_1c_), stimulates the synthesis of IL-10 with the linear stimulation coefficient (S__IL-10_).

The subsequent part of the model accounts for hepatitis progression. The equations describing the time courses of ALT and AST activities in the serum are as follows:

(15) $$\frac{{\mathrm{dA}}_{\mathrm{ALT}}}{dt}=F_{\mathrm{CYT}\_\mathrm{ALT}}\times k_{\mathrm{in}\_\mathrm{ALT}}-k_{\mathrm{out}\_\mathrm{ALT}}\times A_{\mathrm{ALT}},{\;A}_{\mathrm{ALT}}\left(0\right)=R_{0\_\mathrm{ALT}},$$

(16) $$F_{\mathrm{CYT}\_\mathrm{ALT}}=(1+{\left(C_{\mathrm{TNF}-\alpha }-R_{0\_\mathrm{TNF}-\alpha }\right)}^{\alpha }\times S{\_}_{\mathrm{ALT}\left(\mathrm{TNF}-\alpha \right)})\times (1+{\left(C_{\mathrm{IFN}-\gamma }-R_{0\_\mathrm{IFN}-\gamma }\right)}^{\beta }\times S{\_}_{\mathrm{ALT}\left(\mathrm{IFN}-\gamma \right)}),$$

(17) $$\frac{{\mathrm{dA}}_{\mathrm{AST}}}{dt}=F_{\mathrm{CYT}\_\mathrm{AST}}\times k_{\mathrm{in}\_\mathrm{AST}}-k_{\mathrm{out}\_\mathrm{AST}}\times A_{\mathrm{AST}},{\;A}_{\mathrm{AST}}\left(0\right)=R_{0\_\mathrm{AST}},$$

(18) $$F_{\mathrm{CYT}\_\mathrm{AST}}=(1+(C_{\mathrm{TNF}-\alpha }-R_{0\_\mathrm{TNF}-\alpha })\times S{\_}_{\mathrm{AST}\left(\mathrm{TNF}-\alpha \right)})\times (1+(C_{\mathrm{IFN}-\gamma }-R_{0\_\mathrm{IFN}-\gamma })\times S{\_}_{\mathrm{AST}\left(\mathrm{IFN}-\gamma \right)})$$

The disease progression part of the model is composed of two separate indirect response models (Equations (15) and (17)) with stimulatory functions F_CYT_ALT_ and F_CYT_AST_ acting on the zero-order rate constants of ALT and AST production (k_in_ALT_ and k_in_AST_). These stimulatory functions share a similar structure that consists of two independent TNF-α and IFN-γ stimulatory components. Increases in the TNF-α and IFN-γ concentrations above baseline multiplied by the corresponding stimulation coefficients (S__ALT(IFN-γ)_, S__ALT(TNF-α)_, S__AST(IFN-γ)_, and S__AST(TNF-α)_) are driving forces causing elevations in transaminases. The implementation of power functions with α and β exponents was necessary to better describe the influence of TNF-α and IFN-γ on ALT production (Equation (16)). On the other hand, linear functions were sufficient to account for the impact of inflammatory mediators on AST production (Equation (18)). From Equations (15)–(18), it is clear that when the TNF-α and IFN-γ concentrations are not higher than the baseline values (R_0_TNF-α_ and R_0_IFN-γ_), the predicted by the model ALT and AST activities do not increase. This assumption is in agreement with previous findings that TNF-α and IFN-γ are key mediators in the progression of ConA-induced hepatitis, since separate administration of either anti-mouse TNF-α antiserum or a mouse IFN-γ receptor-immunoglobulin fusion protein substantially decreased the activities of transaminases and reduced liver damage in this AIH model in mice [24,25].

R_0_TNF-α_, R_0_IFN-γ_, R_0_IL-6_, R_0_IL-10_, R_0_ALT_, and R_0_AST_ are the baseline serum concentrations of TNF-α, IFN-γ, IL-6, and IL-10 and activities of ALT and AST in female BALB/c mice, calculated as the average of levels measured in samples collected from 4 healthy individuals. Parameters k_in___TNF-α_, k_in___IFN-γ_, k_in___IL-6_, k_in___IL-10_, k_in___ALT_, and k_in___AST_ were not estimated, but were calculated from the following equation:

(19) $$k_{\mathrm{in}\_x}={\;R}_{0\_x}\times {\;k}_{\mathrm{out}\_x}\;$$

where R_0_x_ is the baseline response and k_in_x_ and k_out_x_ are the synthesis and degradation rate constants, respectively, for each biomarker. I_max_TNF-α_, I_max_ IFN-γ_, and I_max_IL-6_ were fixed to 1 during the model fitting to the data.

#### 2.5.2. Statistical Analysis

Statistical analysis was conducted in STATISTICA 13 (Tibco Software Inc., Palo Alto, CA, USA). Due to the lack of a normal distribution and/or homogeneity of variance, all data were firstly transformed using a log-transformation (ln(x)) to obtain a normal distribution and homogeneity of variance, which were verified using Shapiro–Wilk’s and Levene’s tests, respectively. Subsequently, comparisons of the groups were performed using the one-way ANOVA followed by Tukey’s multiple comparison post-hoc test. *p* *<* 0.05 was considered statistically significant.

## 3. Results

### 3.1. Comparative Assessment of PDE Inhibitors in ConA-Induced Hepatitis

**Figure 2:**
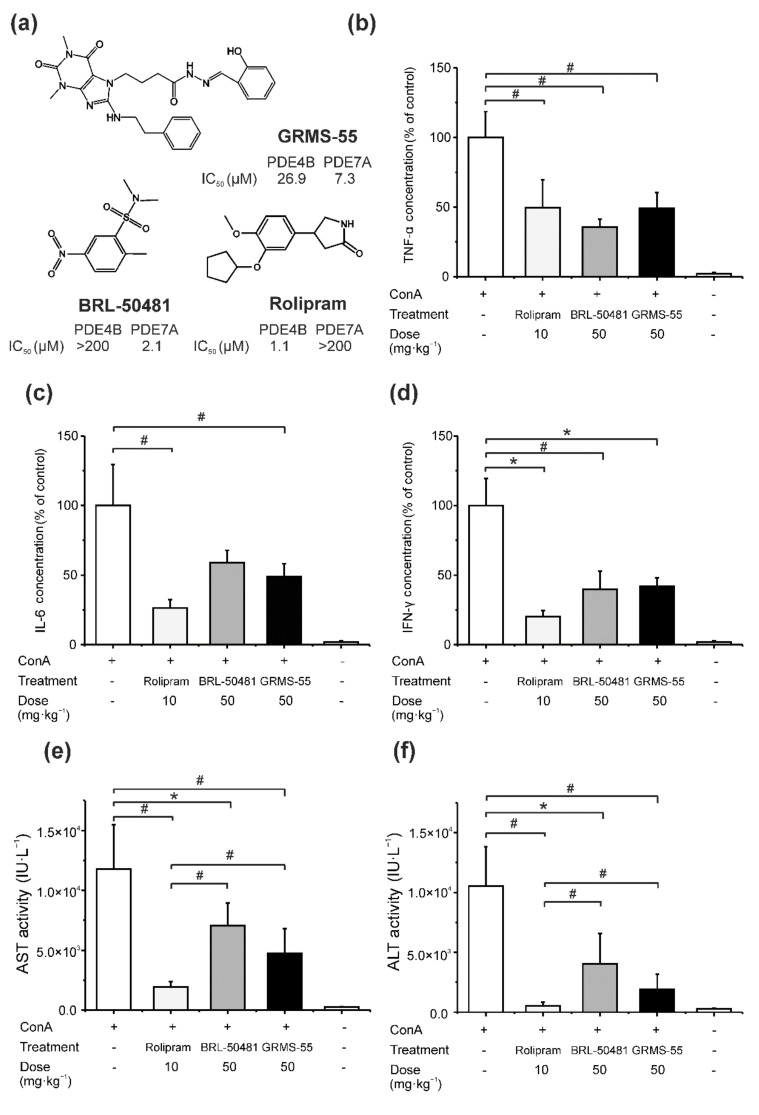
Preliminary pharmacological evaluation of the studied PDE inhibitors in ConA-induced hepatitis. (**a**) Chemical structures of the studied compounds and their IC_50_ values for PDE4B and PDE7A determined in an in vitro assay in our previous work [11]; (**b**–**f**) Effects of the compounds on the levels of inflammatory mediators and transaminases in the serum of mice with ConA-induced hepatitis. Rolipram (10 mg·kg^−1^), BRL-50481 (50 mg·kg^−1^), and GRMS-55 (50 mg·kg^−1^) were administered IP in BALB/c mice 30 min before ConA administration at an IV dose of 20 mg·kg^−1^. Bars represent the mean (+SD) concentrations of (**b**) TNF-α, (**c**) IL-6, and (**d**) IFN-γ and activities of (**e**) AST and (**f**) ALT expressed as % of negative control in the mice serum 8 h following ConA administration (*n* = 6). The negative control group received ConA and vehicle instead of the studied compounds and the healthy control (the last bar in each graph) received saline and vehicle instead of ConA and studied compounds. * *p* < 0.05, # *p* < 0.01, a one-way ANOVA with Tukey’s HSD post-hoc test.

The results of the preliminary pharmacological evaluation are presented in Figure 2. In this experiment, BALB/c mice were pretreated IP with GRMS-55 and two reference compounds, i.e., the PDE7-selective inhibitor BRL-50481 or the PDE4-selective inhibitor rolipram. The negative control group was treated with vehicle alone. Thirty min post-IP dosing, the animals were administered with an IV dose of ConA.

The data presented in the bar charts indicate that all tested compounds significantly decreased the concentrations of the relevant inflammatory mediators compared to the control group except for BRL-50481, which decreased TNF-α and IFN-γ but had no effect on IL-6. Rolipram, which is the strongest PDE4 inhibitor among the tested compounds (Figure 2a), most potently decreased the levels of IFN-γ and IL-6 (approximately 5.0 and 3.8 times, respectively) compared to control group, when administered at a fivefold lower dose than GRMS-55 and BRL-50481. However, no statistically significant differences were observed in the concentrations of the measured inflammatory mediators among the groups of mice treated with GRMS-55, rolipram, and BRL-50481. In contrast, rolipram most strongly reduced the activities of ALT and AST (approximately 18.2 and 6.1 times) compared to the vehicle-treated control group, and this effect was more significant than that observed following BRL-50481 or GRMS-55 administration. The concentrations of TNF-α were decreased in a similar manner by all tested compounds. Taken together, these results imply that selective PDE4 as well as PDE7 inhibition can ameliorate ConA-induced hepatitis. Furthermore, the diminution of IL-6 signaling is not necessarily required to achieve this beneficial effect. This is in line with the previous findings indicating that IL-6 present at the early stage of ConA-induced hepatitis triggers hepatoprotective pathways [26]. On the other hand, the observed reduction in the levels of transaminases seems to be the most strongly associated with a repression of TNF-α and IFN-γ signaling by the studied compounds.

### 3.2. Pharmacokinetics of GRMS-55

**Figure 3:**
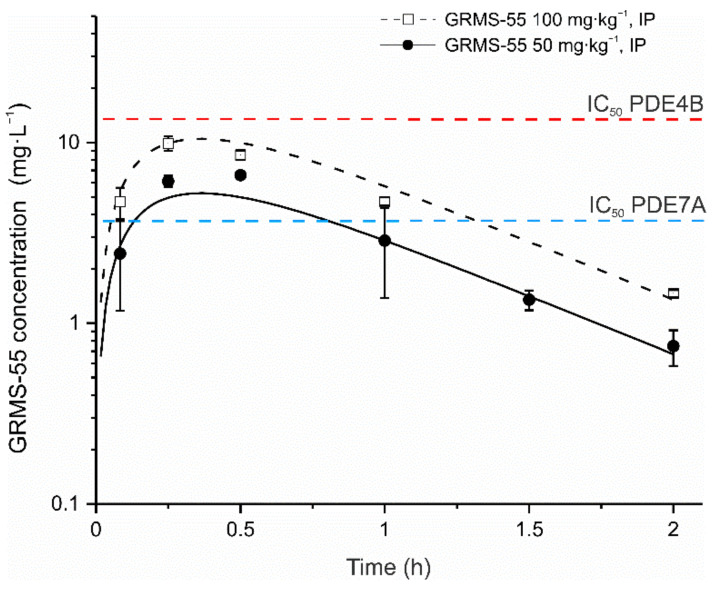
Mean (±SD) concentrations of GRMS-55 in the serum of female BALB/c mice following IP administration of this compound at doses of 50 or 100 mg·kg^−1^ (*n* = 4). Observed concentrations are shown as symbols, and the concentrations predicted by the model are shown as black lines. A blue dashed line indicates the IC_50_ value for PDE7A, and a red dashed line indicates IC_50_ value for PDE4B that were determined in an in vitro assay using GRMS-55 as a PDE inhibitor and reported in our previous publication [11].

GRMS-55 demonstrated a linear pharmacokinetics in female BALB/c mice following IP administration at two dose levels, i.e., 50 and 100 mg·kg^−1^ and a one-compartment model with first-order absorption and first-order elimination satisfactorily described the concentration–time data of the studied compound. The pharmacokinetic model was simultaneously fitted to the data to obtain a single set of pharmacokinetic parameters. The results of model fitting to the experimental data are presented in Figure 3.

As illustrated in the graph, the model predicted concentration vs. time profiles of the investigated compound fairly well captured the levels measured in the mouse serum. The estimated values of pharmacokinetic parameters are listed in Table 1.

From Figure 3 and Table 1, it seems that flip-flop pharmacokinetics following IP administration of GRMS-55 to mice might have occurred. Despite the relatively high value of the k_e_ estimated based on the pharmacokinetic model, the rate of elimination of this compound was limited by approximately a threefold lower rate of its absorption. The serum terminal half-life calculated using the non-compartmental approach (data not shown) was close to 0.5 h, whereas the values of GRMS-55 absorption and elimination half-lives calculated as ln2/k_a_ and ln2/k_e_ were equal to 27.9 and 9.1 min, respectively. However, only the data obtained following IV administration of GRMS-55 in mice may confirm the presumption of the occurrence of flip-flop kinetics. The concentrations of GRMS-55 in the serum of mice in both study groups were above IC_50_ value for PDE7A and below IC_50_ value for PDE4B inhibition. These results suggest a substantial contribution of PDE7A inhibition to the pharmacological effects observed following GRMS-55 administration to mice with ConA-induced hepatitis.

### 3.3. Pharmacodynamics of GRMS-55 and Hepatitis Progression

**Figure 4:**
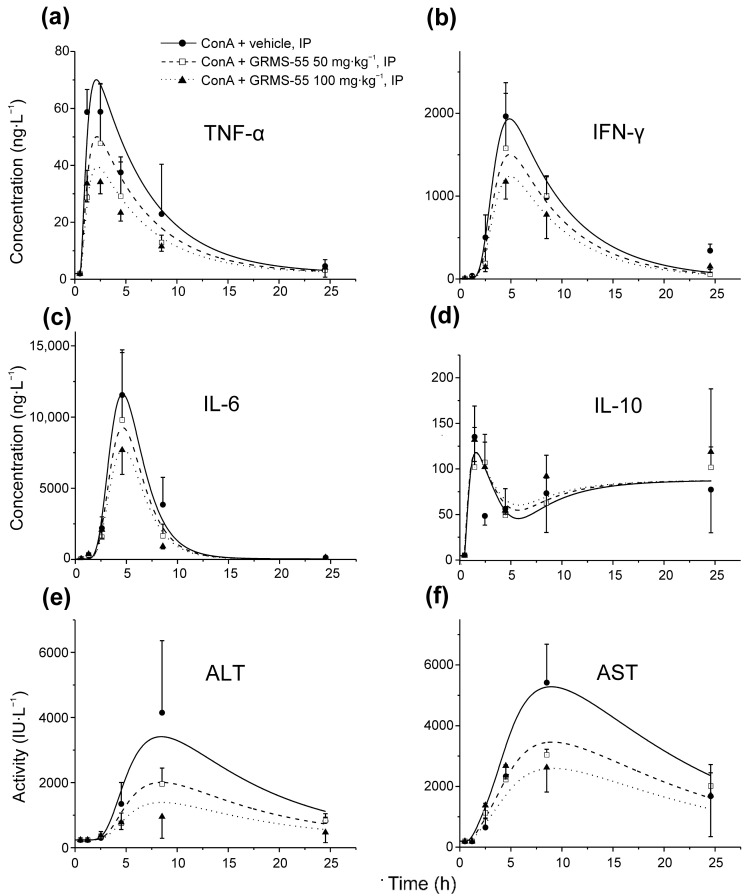
Mean (±SD) concentrations of (**a**) TNF-α, (**b**) IFN-γ, (**c**) IL-6, and (**d**) IL-10 and activities of (**e**) ALT and (**f**) AST in the serum of mice challenged with an IV dose of ConA in the absence or presence of GRMS-55 at doses of 50 or 100 mg·kg^−1^, IP (*n* = 4–5). Observed quantities are shown as symbols, and PK/PD disease progression model predictions are shown as lines.

The time courses of four inflammatory mediators, namely IFN-γ, IL-6, IL-10, and TNF-α and the activities of both transaminases, i.e., ALT and AST, in the mice serum were modeled using the newly designed PK/PD/disease progression model of GRMS-55 in ConA-induced hepatitis in mice. Selection of these biomarkers was made based on the literature data suggesting that TNF-α, IFN-γ, IL-6, and IL-10 are important mediators in the development of ConA-induced hepatitis, and activities of transaminases are reliable indicators of liver damage [13,15]. Secondly, this choice was justified by the fact that the time courses of concentrations of these biomarkers in the serum were, in most cases, captured from the initial increase, throughout the maximum level, to a late stage of the elimination phase. Moreover, the levels of the majority of the selected biomarkers changed in a dose of GRMS-55-dependent manner. Figure 4 shows the observed and model predicted concentrations of IFN-γ, IL-6, IL-10, and TNF-α and the activities of ALT and AST in the serum of mice challenged with an IV dose of ConA in the presence or absence of GRMS-55.

From these graphs, the time courses of the biomarkers were satisfactorily predicted by the proposed PK/PD/disease progression model. Times of their predicted maximal quantities, counted from the moment of ConA challenge, were 1.6, 3.5, 4.4, 8.0, and 8.5 h for TNF-α, IL-6, IFN-γ, ALT, and AST, respectively, and were close to the times of observed maximal levels of these biomarkers presented in the literature [13]. The time course of IL-10 was different when compared to those of other mediators of inflammation as it sharply reached the peak at approximately 1 h and then a decrease in the IL-10 concentration was observed with a minimum at approximately 5 h followed by a gradual increase in this cytokine levels in all tested groups. Pharmacodynamic parameters estimated by the PK/PD model are summarized in Table 1b. From this table, the strongest inhibitory activity of the studied compound was observed in relation to TNF-α as the IC_50_ value of GRMS-55 for inhibition of TNF-α production was more than 1.5 times lower than those for inhibition of either IFN-γ or IL-6. This may suggest that the hepatoprotective effect of GRMS-55 results from its balanced inhibitory activity on the production of inflammatory mediators but is mainly attributed to its TNF-α inhibitory potential. An increase in the levels of TNF-α, IL-6, and IFN-γ has been shown to correlate with the disease severity in ConA-induced hepatitis [15]. However, it must be noted that IL-6 may play a bimodal role in this AIH model by inducing protective pathways and being harmful to hepatocytes [26]. Therefore, it seems that a partial inhibition of TNF-α and IFN-γ could have led to the high hepatoprotective activity, which was observed as a significant and prominent decrease in ALT and AST activities in GRMS-55-treated groups.

### 3.4. Influence of GRMS-55 Administration on the Release of Other Relevant Cytokines in ConA-Induced Hepatitis

**Figure 5:**
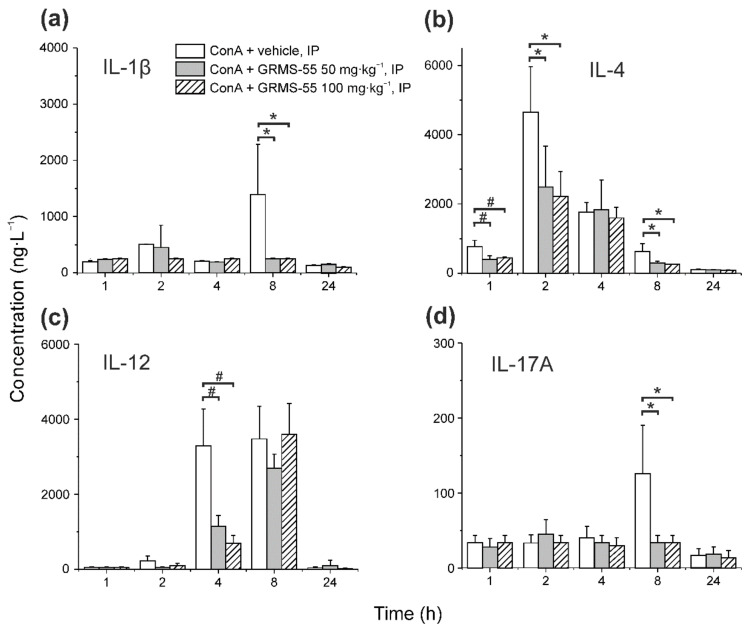
Influence of GRMS-55 administration at two dose levels (50 and 100 mg·kg^−1^, IP) on (**a**) IL-1β, (**b**) IL-4, (**c**) IL-12, and (**d**) IL-17A concentrations in the serum of mice at various time points following IV administration of ConA at a dose of 20 mg·kg^−1^, IV. Each bar represents the mean (+SD) concentration of interleukin in the mouse serum (*n* = 4–5). * *p* < 0.05, ^#^ *p* < 0.01, a one-way ANOVA with Tukey’s HSD post-hoc test.

The levels of several other inflammatory mediators, i.e., IL-1β, IL-4, IL-10, IL-12, and IL-17A, were measured in the serum of mice with ConA-induced hepatitis in the presence and absence of GRMS-55. The results of this analysis are presented in Figure 5.

As it is shown in Figure 5, the concentrations of pro-inflammatory cytokines, i.e., IL-1β and IL-12, were only temporarily increased after ConA administration, and GRMS-55 significantly decreased the levels of IL-12 and IL-1β at 4 and 8 h post-ConA dosing, respectively. This effect was strong but not dose-dependent at the range of doses tested, since there were no significant differences between the concentrations of interleukins between groups treated with the lower and higher dose of GRMS-55. Moreover, the levels of the anti-inflammatory cytokine IL-4 were decreased at 1, 2, and 8 h post-ConA dosing in both GRMS-55-treated groups. An attempt was made to use these data to expand the developed PK/PD model; however, it was unsuccessful, probably due to the lack of significant differences in the concentrations of cytokines between the two groups of mice treated with the GRMS-55 and because of an insufficient number of sampling time points, as in the case of IL-12, which did not allow to capture the complete concentration–time profile of this biomarker. The levels of Il-17A, which is a pro-inflammatory cytokine engaged in the pathogenesis of various autoimmune disorders [27], were increased at 8 h after ConA administration, and GRMS-55 decreased the concentrations of this cytokine at both dose levels.

### 3.5. Assessment of Emetogenicity

**Figure 6:**
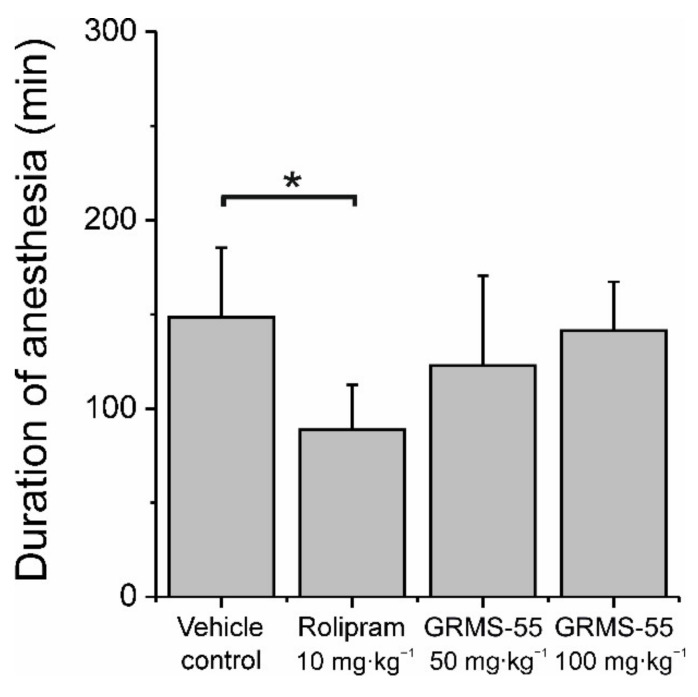
Influence of GRMS-55 administration on the duration of anesthesia induced by an α_2_-adrenoceptor agonist. Bars represent the mean (+SD) time of sleep in each group of mice (*n* = 6); * *p* < 0.05, one-way ANOVA with Tukey’s HSD post-hoc test.

Nausea and vomiting are commonly experienced adverse reactions associated with administration of PDE4 inhibitors. These side effects constitute a major drawback for the clinical development of these compounds. It was suggested that they are related to PDE4D subtype inhibition in the area postrema of the brain stem [28]. The method used to assess the emetogenic potential of PDE inhibitors in non-vomiting species, such as mice, is based on the fact that vomiting induced by PDE4 inhibition is prevented by α_2_-adrenoceptor agonists and, in addition, PDE4 inhibitors prevent α_2_-adrenoceptor agonist-induced loss of the righting reflex and sleep [21]. In the present study, mice were administered IP with the α_2_-adrenoceptor agonist xylazine (10 mg·kg^−1^) and ketamine (80 mg·kg^−1^) that induced the loss of the righting reflex within 5 min post-dosing. The mean (±SD) duration of sleep in the control group was equal to 148.5 ± 36.8 min. The PDE4 inhibitor rolipram at a dose of 10 mg·kg^−1^, IP, significantly shortened the duration of anesthesia by approximately 40% (Figure 6), indicating the emetic-like response.

In the same study, GRMS-55 did not affect the sleeping time at doses up to 100 mg·kg^−1^. These results indicate that GRMS-55 may possess a lower emetogenic potential than rolipram, which makes it a suitable candidate for further development.

## 4. Discussion

This study aimed to assess the efficacy of the PDE inhibitor, GRMS-55, in a mouse model of ConA-induced hepatitis and provide mechanistic considerations regarding the effects of this compound, using mathematical modeling approaches. This animal model is one of the most frequently used AIH models, as it closely resembles the pathogenesis and clinical symptoms of this disease in humans [13,29]. To describe the changes in time of the concentration of inflammatory mediators and the activity of transaminases in mice administered with GRMS-55 at two dose levels or with vehicle alone followed by the ConA challenge, a semi-mechanistic PK/PD/disease progression model was developed.

GRMS-55, a butanehydrazide derivative of purine-2,6-dione, is a strong PDE7A and moderate PDE4B inhibitor. The synthesis, initial pharmacological evaluation, and PK/PD assessment of this compound carried out in animal models of endotoxemia and rheumatoid arthritis were already published by our team [10,11]. In our previous studies, we observed that GRMS-55 and the pan-selective PDE4 inhibitor rolipram exhibited anti-inflammatory effects and decreased paw edema in rats with collagen-induced arthritis, at an IC_50_ of 0.26 and 0.01 mg·L^−1^, respectively, and suppressed TNF-α production in the rat model of lipopolysaccharide-induced endotoxemia at an IC_50_ of 1.06 and 0.36 mg·L^−1^, respectively. These IC_50_ values were estimated using PK/PD arthritis progression and PK/PD models described in one of our previous publications [10]. In addition, we noted that both compounds ameliorated the symptoms of ConA-induced hepatitis in mice and decreased the activities of transaminases and the concentrations of TNF-α in the serum at 8 h following the administration of ConA. The study presented here is a continuation of our previous work on GRMS-55 that mainly focused on performing a thorough assessment of its activity in the ConA-induced hepatitis model.

The concentration–time profiles of GRMS-55 in the serum of mice were well described using a one-compartment model. The terminal half-life of this compound in mice was almost 30 min. The pharmacokinetics of GRMS-55 was previously investigated in male and female rats, and the half-life of this compound in this species was determined to be approximately 20 min. Either a one-compartment or a two-compartment model was found to be relevant to describe the concentration–time profiles of GRMS-55, depending on the sex and strain of the rats [10]. However, this discrepancy could have resulted from sparse blood sampling that might have precluded the observation of a biexponential disposition of GRMS-55 in male rats. Similarly, in the present study, the slow absorption of the compound might have masked the distribution phase. In male Wistar rats, the apparent volume of distribution of GRMS-55 at steady state was considerably higher and close to 7 L·kg^−1^ [10], while in female BALB/c mice the value of this parameter estimated in this study was 1.81 L·kg^−1^. This difference may be partially related to the higher bioavailability of the studied compound in mice compared to that in rats following IP administration. However, the bioavailability was not investigated in the present study.

The results of the preliminary pharmacological evaluation (Figure 2) indicated that rolipram, which is the most potent PDE4 inhibitor among the test compounds, exhibited the highest inhibitory activity, compared to GRMS-55 and BRL-50481, and most strongly decreased the activities of both transaminases. All the test compounds analyzed in the preliminary investigation reduced the concentrations of TNF-α and IFN-γ. In addition, both rolipram and GRMS-55 decreased the levels of IL-6. This observation is in line with the results of previous studies which reported that PDE4 inhibitors diminished the levels of these biomarkers in the ConA-induced hepatitis model [10,30]. Furthermore, in the present study, the strong selective PDE7 inhibitor BRL-50481 reduced the concentrations of both IFN-γ and TNF-α and the activities of transaminases in the ConA-induced hepatitis model; however, the compound did not significantly influence the concentrations of IL-6 at the tested dose. To our knowledge, this study showed for the first time that a selective PDE7 inhibitor effectively ameliorated liver damage in ConA-induced hepatitis. Other PDE7 inhibitors, which were investigated previously in this animal model of AIH, were not selective, but exhibited a significant PDE4-inhibitory activity [10,31]. Due to the relatively high PDE4-suppressing activity of the studied inhibitors, it was not clear if the observed pharmacological effect resulted from PDE7, PDE4, or dual PDE4/7 inhibition. The results presented in this paper imply that a selective diminution of PDE7 activity caused anti-inflammatory and hepatoprotective effects in the ConA-induced hepatitis model and the observed outcomes may be attributed to the suppression of TNF-α and production of IFN-γ. GRMS-55, given at a fivefold higher dose than rolipram, caused a similar reduction in the levels of IFN-γ, TNF-α, and IL-6. It must be noted, though, that GRMS-55 is a 24.5-time weaker inhibitor of PDE4B than rolipram and a 3.5-time weaker PDE7A inhibitor than BRL-50481. Furthermore, GRMS-55 was not found to inhibit PDE4D in an in vitro assay at concentrations up to 200 µM [10], while it reached much lower concentrations in the mouse brain following IP administration at a dose of 50 mg·kg^−1^ (data not shown). Taken together, these results suggest that simultaneous inhibition of both PDE7A and PDE4B induced anti-inflammatory effects in ConA-induced hepatitis by inhibiting the production of pro-inflammatory mediators. Therefore, GRMS-55 may be considered a safer and more efficient alternative to PDE4-selective inhibitors and a promising drug candidate for the treatment of AIH as it possesses anti-inflammatory activity and does not cause side effects related to the inhibition of the PDE4D subtype.

Disease progression modeling is an approach gaining increasing interest as it helps to improve and accelerate the decision-making process during the preclinical and clinical stages of drug development. It also allows us to better describe and understand the mechanisms of action of drugs and investigational compounds, as well as the pathophysiological processes underlying the progression of various disorders. Disease progression models often enable predicting which drugs or investigational compounds may be used to target specific signaling molecules that are responsible for disease progression. The available literature provides many examples of disease progression models used in preclinical studies to mathematically describe the interdependencies among the pharmacokinetics of the studied compounds, their pharmacodynamic responses, and changes in the time of disease endpoints. For instance, these models were used for the assessment of anti-inflammatory drugs in a collagen-induced arthritis model in rats, taking into account the interrelations among the inflammatory mediators and their influence on bone density and paw edema [32,33]. To our knowledge, the PK/PD/disease progression model developed in the present study is the first attempt to mathematically describe ConA-induced hepatitis in mice, which is the most frequently used animal AIH model. In addition, for the first time, the effects of an investigational compound in this animal model of AIH were mathematically described in the study. It is a semi-mechanistic model, which reflects the multistep pathogenesis of immune-mediated hepatitis and also provides insights into the mechanism of action of GRMS-55. The pathogenesis of ConA-induced hepatitis has been thoroughly studied and described in the literature, with division into several phases. Firstly, the intravenous administration of ConA induces lymphoid cells, such as macrophages and CD4+ T helper (Th) cells, to release TNF-α, considered the first cytokine and the level of which reaches the peak value as early as 1–2 h post-ConA challenge. Upon activation, Th cells produce several inflammatory mediators, such as IFN-γ, IL-4, and IL-6. It was noted that CD4+ Th cells recognize the ConA-modified major histocompatibility complex structures on macrophages and become activated triggering an inflammatory reaction and the release of interleukins [13]. Subsequently, neutrophils and T cells infiltrate into the liver [15]. By using transit compartments and indirect response models, the PK/PD/disease progression model presented in this study allowed capturing the delays in the production and release of inflammatory mediators and transaminases, as well as describing the interdependencies among them. In the pharmacodynamic part of the model, the first equation (Equation (3)) accounts for the dynamics of the first hypothetical mediator, which appears immediately following the administration of ConA (T_1_) and is a starting point of the model and a part of all other transduction pathways that lead to the build-up of pharmacodynamic responses. This approach allows reducing the number of parameters, improving the precision of parameter estimates, and dealing with the issue of identifiability. The limitation of this study is the lack of a ConA concentration–time profile, which could have been used instead of the first empirical hypothetical mediator. Therefore, future studies should measure the concentrations of this lectin in the blood and/or tissues of mice for a deeper understanding of the mechanisms of disease development in the initial phase. The main assumption of the PK/PD model developed in this study is that GRMS-55 acts at the early stage of the disease by inhibiting the production of the hypothetical precursors of inflammatory mediators. This is in accordance with the actual mechanism of action of PDE inhibitors, which are known to suppress the gene transcription of various pro-inflammatory cytokines and other signaling molecules. This activity may be related to the increase in cAMP concentrations in immune cells via the inhibition of PDE4B and/or PDE7A, which are abundantly present in different types of white blood cells [6]. The rise in intracellular cAMP concentration leads to an increase in the phosphorylation of PKA, resulting in subsequent phosphorylation of transcription factors that directly regulate the expression of genes coding for inflammatory mediators, including cytokines and interferons [34]. In the pharmacodynamic part of the mathematical model presented in this study (Figure 1b), the inhibitory function, which is known from the literature as the I_max_ model, was implemented in the first equation in each series of transit compartments (Equations (4), (6) and (9)). In Equation (3), the input (at 0.5 h post-GRMS-55 dosing) was set to 1 for simplicity, so the initial signal is the same for each pathway. In turn, the output signal in each series of transit compartments was multiplied by a stimulation coefficient in the final equations (Equations (5), (8), (11) and (13)) to account for the production of TNF-α, IFN-γ, IL-6, and IL-10, respectively. This approach allows the use of absolute instead of normalized concentration values for each biomarker.

The IC_50_ values estimated using the PK/PD model (Table 1b) indicate that GRMS-55 most strongly suppressed the production of TNF-α and weakly inhibited IFN-γ and IL-6. The IC_50_ values of GRMS-55 for the inhibition of TNF-α, IFN-γ, and IL-6 were 7.97, 12.27, and 13.40 µM, respectively, while the values for PDE7A and PDE4B inhibition reported in our previous publication were 7.3 and 26.9 µM, respectively [10]. Moreover, the concentrations of GRMS-55 achieved in the serum of animals in both study groups were below the IC_50_ value for PDE4B, but above that for PDE7A inhibition (Figure 3). These results imply that PDE7A suppression significantly contributed to the overall pharmacological effect of GRMS-55 in ConA-induced hepatitis. Considering that the results of the present study as well as the literature data indicate the main influence of TNF-α and IFN-γ on liver damage in ConA-induced hepatitis, it can be concluded that GRMS-55 exhibits a hepatoprotective activity primarily by inhibiting the production of TNF-α and, to a lesser extent, by decreasing IFN-γ signaling. Furthermore, it seems that these effects of GRMS-55 are mainly related to PDE7A suppression, whereas PDE4B inhibition plays a smaller role. Based on the data obtained, we were able to mathematically describe the interplay between the studied inflammatory mediators, i.e., the inhibitory effect of IFN-γ on the production of IL-10, which is in accordance with the results of earlier studies [23]. Despite several attempts, the other interrelations among the studied signaling molecules could not be captured. However, the assessment of the local concentrations of cytokines and measurement of their expression in liver tissue in future studies may allow for further development of this model. The elimination half-life values of most of the studied biomarkers, calculated as ln2/k_out,_ were reasonable and close to those reported in the literature. The half-life value of ALT and AST estimated in this study was 6.95 and 8.48 h, respectively, while that of ALT in mice, reported in the literature, was 4 h [35]. In the case of IFN-γ, the elimination half-life estimated in this study was 2.96 h, and the value determined in the previous investigation in mice was 2.16 h [36]. The half-life value of IL-6 estimated in this study was 1.31 h, while that reported in humans was 1.72 h [37]. In turn, the half-life value estimated for TNF-α was 3.32 h, while the literature data indicate a shorter half-life for this cytokine of up to 30 min in rodents and approximately 70 min in humans [37,38]. This discrepancy may arise from different calculation approaches, experimental conditions, and methods of quantification. Liver injury is associated with an increased activity of ALT and AST in serum, of which ALT is more specific to liver cell death, while the activity of AST in serum may also increase during heart or skeletal muscle damage. The levels of transaminases start to increase in the blood not earlier than 1.5–3 h post-ConA injection. In the mathematical model developed in this study, we assumed that changes in the transaminase activities in the mouse serum reflect the progression of hepatitis and that both TNF-α and IFN-γ contribute to liver damage. Among the alternative models tested, assuming the influence of other inflammatory mediators on disease progression, none gave better results than the final model described in this paper.

In the present study, it was observed that all alterations in the levels of inflammatory mediators and biomarkers resulting from ConA administration were at least partially ameliorated by GRMS-55. The possible mechanism of action of this PDE inhibitor is most likely related to its ability to increase the concentration of intracellular cAMP in immune cells, such as T and B lymphocytes, monocytes, macrophages, neutrophils, and others in which PDE4B and PDE7A are abundantly present [6]. This pathway, as mentioned earlier, regulates the gene expression of inflammatory mediators [34]. Therefore, following the administration of PDE4 and PDE7 inhibitors, the levels of pro-inflammatory mediators, such as TNF-α and IFN-γ, decrease. Moreover, previous studies showed that the inhibition of PDE4, as well as PDE7, affects the T cell proliferation, which can also contribute to the overall pharmacological effect of GRMS-55 [39,40]. Following ConA administration in mice, a cascade of immune responses is triggered, leading to the production and release of inflammatory mediators, recruitment of immune cells (especially Th cells), and subsequent liver injury [13]. The present study revealed a similar pattern of release of inflammatory mediators and transaminases as previous studies on ConA-induced hepatitis in mice [20,41,42]. The levels of IL-12 were observed to reach the peak value between 4 and 8 h, while the concentrations of the anti-inflammatory cytokine IL-10 peaked at 1.1 h post-ConA dosing, then decreased until 5.1 h, and gradually increased reaching a new steady-state concentration at 24 h. This complicated time course of IL-10 may be partially explained by the interrelations among signaling molecules. In our study, an incorporation of IFN-γ influence on the rate of IL-10 production improved the model fitting. This assumption is in line with the results of earlier in vitro studies in which IFN-γ suppressed both IL-10 gene expression and protein concentrations in LPS-stimulated human monocytes [23]. Nonetheless, extended sampling is required in future experiments to explore the changes in IL-10 concentration at later time points. In this study, the concentrations of pro-inflammatory cytokines, IL-1β and IL-17A, increased in the ConA-treated control group at 8 h post-mitogen injection, but this effect was completely diminished in both groups treated with GRMS-55. IL-17A is specifically produced by Th17 cells, which are closely associated with the pathogenesis of autoimmune disorders. The concentrations of this interleukin can be found increased in subjects with AIH, rheumatoid arthritis, and multiple sclerosis [27,43]. The results of earlier studies suggest that the inflammatory mediators IFN-γ, TNF-α, and IL-6 play a critical role in ConA-induced hepatitis as well as in the development of AIH in humans [15,44]. TNF-α mediates liver injury by inducing the apoptosis of hepatocytes. A study showed that IFN-γ-deficient mice have a significantly reduced liver injury due to a decreased expression of Fas, which is the receptor contributing to the apoptosis of hepatocytes [45]. It was noted that IL-6 may display a hepatoprotective effect when released at an early stage of disease development, while its elevated concentrations at a later phase may be harmful to hepatocytes [26]. Therefore, an increase in the levels of this cytokine may not directly correlate with the extent of liver injury in this animal AIH model. The PK/PD/disease progression model developed in this study assumed the joined effect of IFN-γ and TNF-α on the production of transaminases. The results of previous studies suggest that IL-10 ameliorates the symptoms of ConA-induced hepatitis [13]. The present study assumed that IL-10 has no effect on disease development, as the model developed in this study exhibited the best goodness-of-fit criteria compared to the alternative models assuming the influence of IL-6 or IL-10 on disease progression. However, other mediators may also contribute to the progression of hepatitis; therefore, the presented PK/PD/disease progression model should be further developed and include the assessment of the expression of other inflammatory cytokines and especially chemokines at the gene and protein levels as well as other biomarkers of liver damage (gamma-glutamyl transpeptidase and alkaline phosphatase), liver function (e.g., bilirubin concentration in the blood or prothrombin time), and liver fibrosis. It should be mentioned that this model has some limitations as it does not consider the possible threshold levels of inflammatory mediators of clinical importance or include subsequent signaling pathways that are triggered at a cellular level. Nonetheless, the PK/PD model developed in this study may be useful as such in future studies for assessing the activity of not only PDE inhibitors but also other anti-inflammatory and immunomodulatory compounds as potential drug candidates for the treatment of AIH.

Some selective PDE4 inhibitors have already been approved for the treatment of inflammatory and autoimmune disorders. For instance, apremilast and roflumilast are clinically used for treating psoriatic arthritis and chronic obstructive pulmonary disease, respectively. However, administration of these compounds leads to gastrointestinal side effects, among which the most undesirable are nausea and vomiting. These adverse reactions are believed to be resulting from the inhibition of PDE4D in the area postrema of the brain stem [46]. Several strategies are applied in the discovery and development of new PDE inhibitors, with the aim of reducing these side effects. These include designing selective PDE4B inhibitors, allosteric PDE4B modulators, and dual PDE4/7 inhibitors [6,7,47]. The latter approach assumes that compounds that inhibit both PDE4 and PDE7 may possess stronger anti-inflammatory and immunomodulatory properties resulting from the synergy, and thus may be used at lower doses, compared to selective PDE4 inhibitors, to achieve the desired therapeutic effects. GRMS-55 possesses strong PDE7A-inhibitory and moderate PDE4B-inhibitory properties, and is not active against PDE4D at concentrations up to 200 µM. Moreover, it suppresses PDE1B and PDE3A with high and moderate potency, respectively [10]. Therefore, this compound seems to have a well-balanced inhibitory profile against different types and subtypes of PDEs, which minimizes the risk of potential side effects. To verify the possible superiority of GRMS-55 over a strong pan-selective PDE4 inhibitor in terms of potential adverse reactions, we investigated the time shortening of α_2_-adrenoceptor agonist-induced loss of the righting reflex by PDE4 inhibitors. It is postulated that these compounds trigger emesis by mimicking the pharmacological action of α_2_-adrenoceptor antagonists as they can reverse the hypnotic effect of α_2_-adrenoceptor-mediated anesthesia in rats and ferrets [21]. In this study, we found that rolipram significantly reduced the duration of anesthesia induced by the administration of xylazine and ketamine, while GRMS-55 did not influence the time of sleep at doses up to 100 mg·kg^−1^. These results and the previously published data on the PDE selectivity of GRMS-55 imply that this compound may possess reduced side effects (i.e., nausea and emesis) compared to the reference compound, rolipram, which despite proving its high efficacy in preclinical studies has not been registered by any regulatory agency so far, mainly due to the above-mentioned adverse reactions.

## 5. Conclusions

The results presented in this paper indicate that due to the balanced anti-inflammatory properties, resulting mainly from PDE7A and, to a lesser extent, PDE4B inhibition, GRMS-55 exhibited high hepatoprotective activity in mice with ConA-induced hepatitis. This effect was mostly mediated by the suppression of the production of pro-inflammatory mediators, namely TNF-α and IFN-γ. Moreover, enhanced anti-inflammatory signaling was observed in the GRMS-55-treated groups compared to the control group, which was manifested by elevated concentrations of IL-10; however, according to the model predictions and the literature data, this phenomenon seems to be directly associated with concentration-dependent inhibition of IL-10 production in the presence of IFN-γ. The progression of hepatitis is driven mainly by TNF-α and IFN-γ, which simultaneously contribute to the death of liver cells. GRMS-55 may display potentially lower emetogenicity compared to the potent PDE4-selective inhibitor, rolipram, probably due to its PDE4B selectivity over PDE4D, the inhibition of which is believed to be responsible for adverse reactions, such as nausea and emesis, which are commonly observed following the administration of PDE4 inhibitors. Therefore, GRMS-55 is a promising drug candidate for the treatment of AIH as it possesses anti-inflammatory activity and does not cause any side effects related to the inhibition of the PDE4D subtype. Based on the results obtained, it can be concluded that dual PDE4/7 inhibitors may constitute a safer therapeutic alternative compared to the standard immunosuppressive drugs used for the treatment of AIH as well as other inflammatory and autoimmune disorders. A novel semi-mechanistic PK/PD/disease progression model was developed in this study, accounting for the delayed action of GRMS-55, interdependencies among relevant signaling molecules, and the joined effect of pro-inflammatory mediators on disease development in ConA-induced hepatitis in mice. This model may be used as such in future studies to assess the activity and mechanisms of action of new investigational compounds for the treatment of AIH. In addition, the model may be extended further by involving other biomarkers of pharmacodynamic response and/or disease progression and by incorporating physiologically–based pharmacokinetic models.

## Figures and Tables

**Table 1:** Values of parameters of the PK/PD disease progression model. (**a**) Pharmacokinetic, (**b**) pharmacodynamic, and (**c**) disease progression parameters of GRMS-55 administered to BALB/c mice at single IP doses of 50 or 100 mg·kg^−1^. Mice in the PD/disease progression study were challenged with ConA at an IV dose of 20 mg·kg^−1^ 30 min following GRMS-55 administration.

Analysis	Parameter	Brief Description	Final Estimate	CV (%)
(a) Pharmacokinetic	Vd/F (L·kg^−1^)	Apparent volume of distribution of GRMS-55	1.81	32
	k_a_ (h^−1^)	Absorption rate constant of GRMS-55	1.49	21
	k_e_ (h^−1^)	Elimination rate constant of GRMS-55	4.59	30
(b) Pharmacodynamic	τ (h)	Mean transit time	0.405	9
	k_out_IFN-γ_ (h^−1^)	Elimination rate constant of IFN-γ	0.234	24
	k_out_IL-6_ (h^−1^)	Elimination rate constant of IL-6	0.531	17
	k_out_TNF-α_ (h^−1^)	Elimination rate constant of TNF-α	0.209	17
	k_out_IL-10_ (h^−1^)	Elimination rate constant of IL-10	1.841	57
	IC_50_IFN-γ_ (mg·L^−1^)	GRMS-55 concentration resulting in 50% of I_max_ of IFN-γ	12.27	24
	IC_50_ IL-6_ (mg·L^−1^)	GRMS-55 concentration resulting in 50% of I_max_ of IL-6	13.40	28
	IC_50_TNF-α_ (mg·L^−1^)	GRMS-55 concentration resulting in 50% of I_max_ of TNF-α	7.97	21
	S__IFN-γ_	IFN-γ synthesis stimulation coefficient	5856	17
	S__TNF- α_	TNF-α synthesis stimulation coefficient	453.9	16
	S__IL-6_	IL-6 synthesis stimulation coefficient	10,320	13
	S__IL-10_	IL-10 synthesis stimulation coefficient	2.035	64
	k_in___IL-10(dis)_ (ng·L^−1^·h^−1^)	Production rate constant of IL-10 in diseased animals	160.8	61
	I__ IL-10(IFN-γ)_ (L·ng^−1^)	IL-10 synthesis inhibitory coefficient	0.0002	36
(c) Disease progression	S__ALT(TNF-α)_	Stimulation coeffcient of ALT production by TNF-α	0.0072	84
	S__ALT(IFN-γ)_	Stimulation coeffcient of ALT production by IFN-γ	0.0002	48
	k_out_ALT_ (h^−1^)	Elimination rate constant of ALT	0.0997	28
	S__AST(TNF-α)_ (L·ng^−1^)	Stimulation coeffcient of AST production by TNF-α	0.7727	37
	S__AST(IFN-γ)_ (L·ng^−1^)	Stimulation coeffcient of AST production by IFN-γ	0.0007	83
	k_out_AST_ (h^−1^)	Elimination rate constant of AST	0.0817	20
	α	Power coeffcient for stimlation of ALT production by TNF-α	1.5 ^a^	–
	β	Power coeffcient for stimlation of AST production by IFN-γ	1.5 ^a^	–

^a^ Fixed value.

## Data Availability

The data presented in this study are available on request from the corresponding author.
